# Supplementary material for: Pulsed transistor operation enables miniaturization of electrochemical aptamer-based sensors
Source: Sci Adv. 2022 Nov 16;8(46):eadd4111. doi: 10.1126/sciadv.add4111 (PMC9668304; doi:10.1126/sciadv.add4111)
Supplement: Supplementary file 1 — Figs. S1 to S7 [file sciadv.add4111_sm.pdf]

Science Advances
AAAS

Supplementary Materials for

Pulsed transistor operation enables miniaturization of electrochemical aptamer-based sensors

Sophia L. Bidinger *et al.*

Corresponding author: George G. Malliaras, gm603@cam.ac.uk; Tawfique Hasan, th270@cam.ac.uk

Sci. Adv. **8**, eadd4111 (2022)
DOI: 10.1126/sciadv.add4111

This PDF file includes:

Figs. S1 to S7

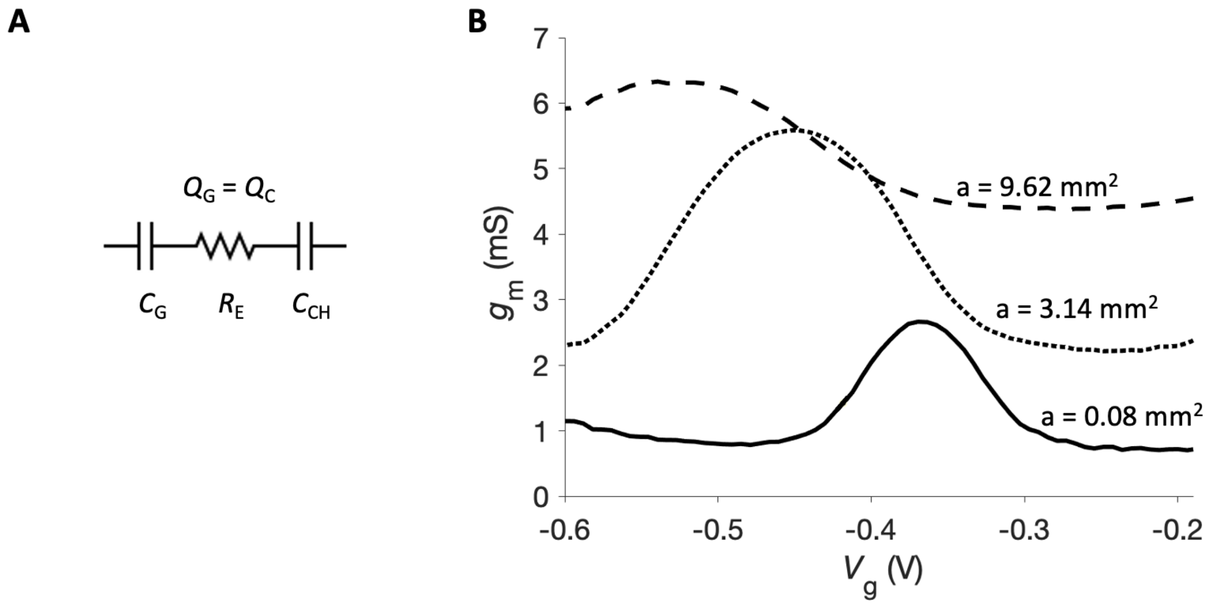

Fig. S1. Optimizing device geometry.
(A) The equivalent circuit representing gate and channel capacitance (C_G and C_{CH}) with electrolyte resistance (R_E). **(B)** DC transconductance for various gate electrode areas using the same channel area (W = 50 μm, L = 400 μm). The methylene blue redox peak shifts to lower voltages as gate area increases resulting in more voltage drop at the channel side. The capacitance of a representative electrode with a = 3.14 mm^2 is 10.6 μF with a corresponding channel capacitance of 33.4 μF, measured with an R-CPE circuit fit from electrochemical impedance spectroscopy. Of note, both the device geometry and the drain voltage magnitude affect the redox peak position, which is effectively being referenced against the PEDOT:PSS channel (*5*).

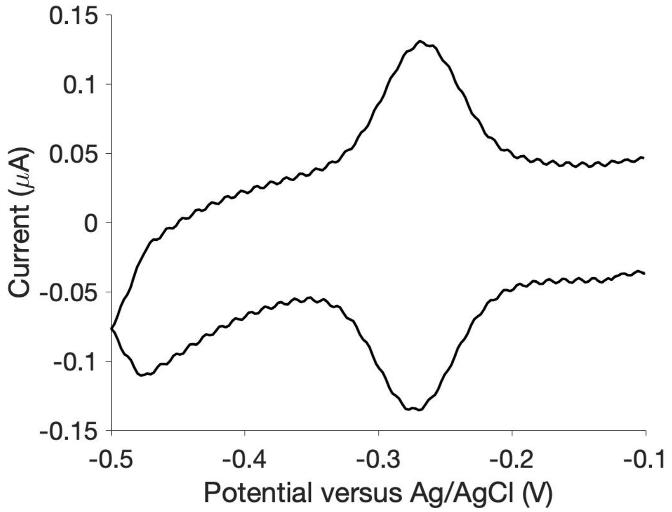

Fig. S2. Determination of aptamer packing density with cyclic voltammetry.
Cyclic voltammogram of an EAB electrode. The number of aptamers on an electrode can be determined using the methylene blue oxidation peak area, as each molecule contributes two electrons per interrogation scan. This is divided by the electrode area to determine the aptamer packing density.

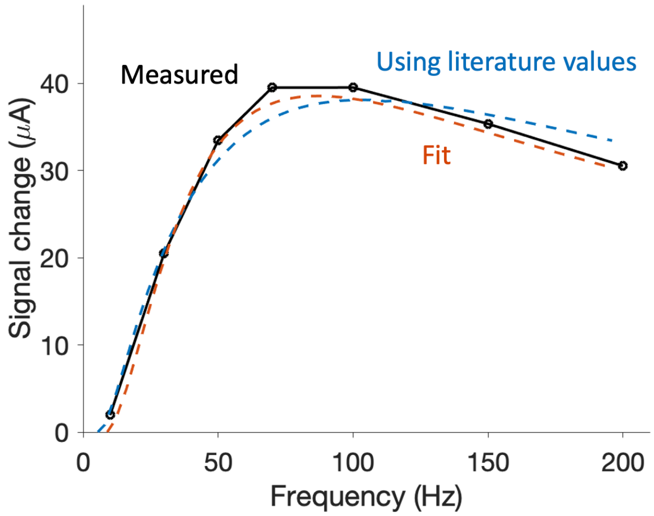

Fig. S3. Comparison of measured and simulated AB-OECT frequency response.
Measured SWV OECT signal gain upon 500 μM tobramycin addition (solid black line) versus expected gain from a basic simulated AB-OECT models (dashed lines). The blue dashed line represents a model using k_{bound} and k_{unbound} values for the aminoglycoside aptamer from previous literature *(14)* as well as established OECT governing equations *(19)* and the measured aptamer site density. The expected response is calculated from a simplified single square pulse. The

orange dashed line corresponds to the same model fit to the measured data, which returns time constants of $k_{unbound}$ = 22.3 ms and k_{bound} = 6.70 ms.

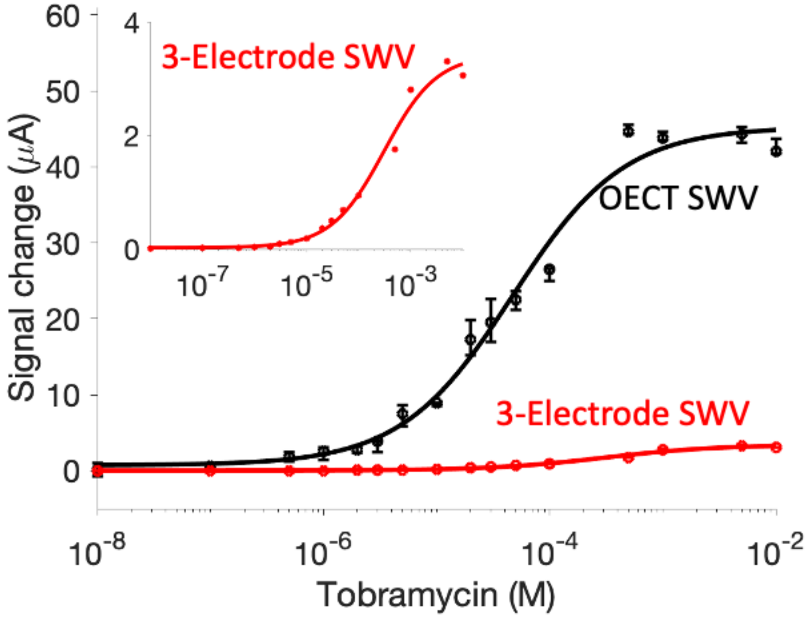

Fig. S4. Measurement variation.
Binding curves of the AB-OECT and EAB sensors using identically-sized gate and working electrodes measured in PBS. Error bars correspond to the standard deviation of three consecutive scans collected at each concentration.

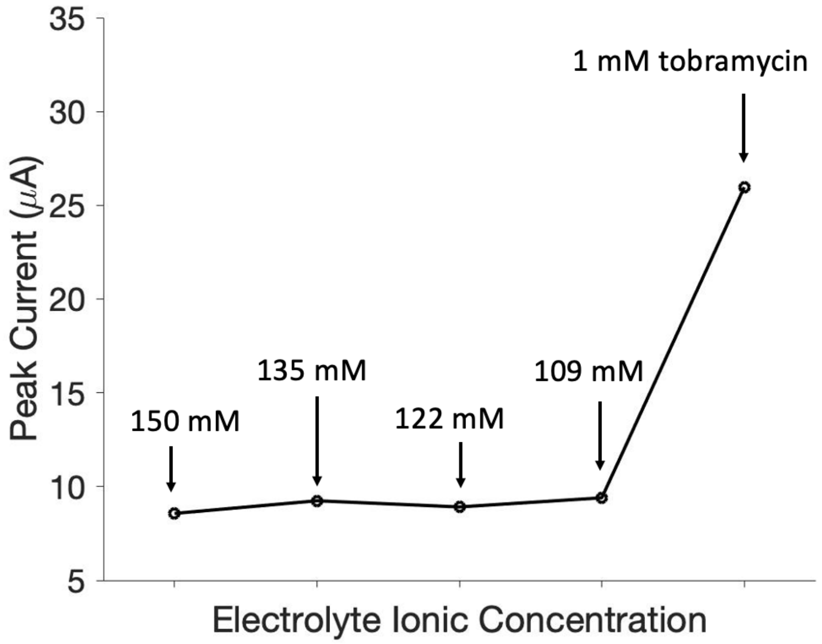

Fig. S5. AB-OECT ionic selectivity.
The response of an AB-OECT sensor is largely independent of ionic strength. Shown here, for example, are sequential dilutions from 1x PBS electrolyte (150 mM ionic strength, which similar to that of blood) followed by spiking with 1 mM tobramycin. This relative insensitivity to ionic strength is a significant advantage of pulsed operation for OECT-based sensing relative to DC techniques for in-vivo translation, where the ionic environments is prone fluctuate.

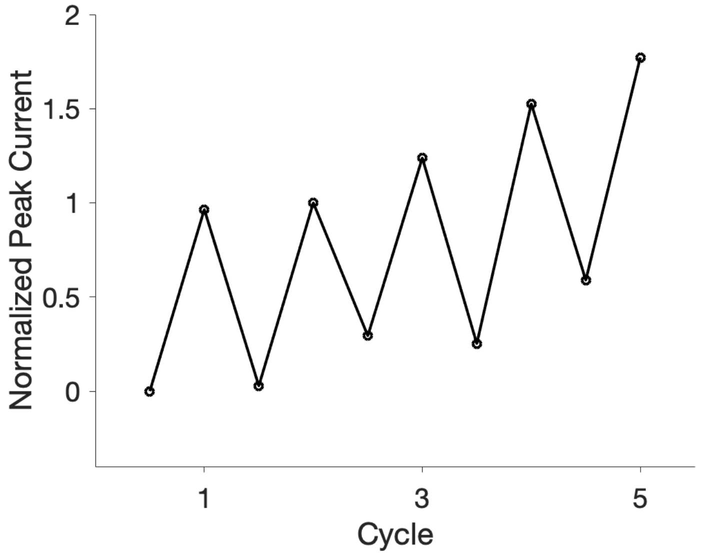

Fig. S6. Reversibility in whole blood. Consecutive spike and rise cycles in whole blood yield consistent peak current reversibility, albeit with some current drift.

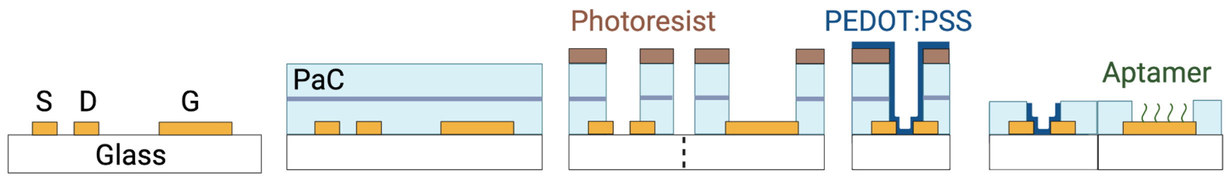

Fig. S7. AB-OECT fabrication protocol.
We begin with gold source (S), drain (D), and gate (G) electrode patterning on glass substrates. Next, two parylene-C (PaC) layers are deposited with an anti-adhesive between for peel-off. A second lithography step is used to expose the channel and gate areas then the glass substrates are diced to separate gates and channels. PEDOT:PSS is spin-coated over the channels and the aptamer is immobilized on the gate. The gates and channels are reconnected with a well for testing.

Supplementary Text 1. Modelling of the AB-OECT response.

The response of the AB-OECT is estimated by estimating the gate current transient and using Eqn. [1] from the main text to predict the output modulation in drain current ΔI_D. First, the fraction of bound aptamer (f_b) sites was calculated using the dissociation constant ($K_d = 1 \times 10^{-5}$). *(6)*

$$\boldsymbol{f_b = \frac{C_{TOB}}{C_{TOB}+K_d}} \text{ [S1]}$$

Where C_{TOB} is the concentration of tobramycin in solution. Then, we take the total gate current I_G as a function of time (t) as the sum of the double layer charging ($\boldsymbol{I_{DL}}$), and oxidation currents from bound ($\boldsymbol{I_b}$) and unbound ($\boldsymbol{I_u}$) aptamers at the surface.

$$\boldsymbol{I_G(t) = I_{DL}(t) + I_b(t) + I_u(t)} \text{ [S2]}$$

The currents due to oxidation of methylene blue (MB) from bound and unbound aptamer sites are calculated as exponential decays using with characteristic electron transfer time constants of k_b and k_u for bound and unbound aptamers, respectively, in Eqns. S3 and S4 below.

$$\boldsymbol{I_b(t) = k_b f_b 2eC_{MB}Aexp(-tk_b),\; I_u(t) = k_u(1-f_b)2eC_{MB}Aexp(-tk_u)} \text{ [S3, S4]}$$

Where, C_{MB} is the surface density of methylene blue in cm^{-2}, e is the elementary charge, and A is the electrode area. The current from double layer charging can be estimated with Eqn. S5 below.

$$\boldsymbol{I_{DL}(t) = \frac{\Delta V}{R_{eq}} * exp\left(\frac{-t}{R_{eq}C_{eq}}\right)} \text{ [S5]}$$

Where ΔV is the applied pulse voltage, R_{eq} and C_{eq} are the resistance and capacitance of the equivalent device circuit described in Fig. S1A. For a fixed device geometry, I_{DL} is independent

of concentration, so the increase charge carrier modulation ΔQ for a given frequency (F) and concentration will be determined by Eqn. S6 below.

$$\boldsymbol{\Delta Q(F, f_b) = \int_{t=0}^{1/F} (I_b(t, f_b) + I_u(t, f_b))dt - \int_{t=0}^{1/F} I_u(t, f_b = 0)dt} \text{ [S6]}$$

Finally, the output signal change from the OECT can be calculated using the Eqn. 1 and substituting the integrated gate current for the charge modulation ΔQ in Eqn. S7 follows:

$$\Delta I_D(f, f_b) = \frac{\mu}{L^2} V_D * \Delta Q(F, f_b) \text{ [S7]}$$

The simulated frequency dependent response was produced by using a hole mobility of $\mu = 1.2$ $cm^2 V^{-1} s^{-1}$. The blue curve used literature values of $k_b = 250 s^{-1}$, $k_u = 30.3 s^{-1}$, and the measured aptamer site density $D = 1.22 \times 10^9 mm^{-2}$. The orange curve optimized k_b, k_u, and D to fit the experimental data, giving values of 149 s^{-1}, 44.8 s^{-1}, and $1.96 \times 10^9 mm^{-2}$, respectively.
